# Impacts of land consolidation in a semi-arid agricultural region of Türkiye using remote sensing analysis

**DOI:** 10.1007/s10661-026-15195-3

**Published:** 2026-03-22

**Authors:** Furkan Yilgan, Sedat Dogan

**Affiliations:** 1 Faculty of Agrobiology, Food and Natural Resources, Department of Soil Science and Soil Protection, Czech University of Life Sciences Prague, Kamýcká 129, Praha-Suchdol, 165 00 Prague Czech Republic; 2 Faculty of Engineering, Department of Geomatics Engineering, Ondokuz Mayis University, Körfez, No. 64, Atakum, 55270 Türkiye

**Keywords:** Google earth engine, Landsat, Rural development, Sentinel-1, Sustainable land management, Vegetation stress

## Abstract

Land consolidation (LC) activities are important for sustainable agricultural management, as they help improve irrigation, create more productive agricultural parcels, and increase yields. Combining small irregular parcels into larger, regular soil plots enables more efficient and sustainable agricultural practices. Fragmented land reduces productivity and contributes to irrigation inefficiency and soil erosion. This study analyzed the impact of LC on vegetation cover and moisture retention in agricultural land parcels across four villages in the Kızıltepe District of Mardin Province, which is located within the GAP region of Türkiye. This study evaluated changes in agricultural land parcels between 2015 and 2019 (pre-LC) and 2020 and 2024 (post-LC) for three different months (April, May, and June) using Sentinel-1 synthetic aperture radar (SAR) and Landsat-8/9 remote sensing datasets. Changes in the normalized difference vegetation index (NDVI), the normalized difference moisture index (NDMI), and the modified normalized difference water index (MNDWI) were observed, and their relationships with the SAR change data were checked using 944-pixel samples by Pearson’s correlation coefficients. NDVI and NDMI changes showed strong positive correlations (> 0.70), while MNDWI changes showed weak correlations with Sentinel-1 SAR data for all 3 months. The results show that LC has positively affected agricultural productivity, particularly by improving water retention and plant health at the beginning of the agricultural season. Significant positive changes were seen in April. These indicate more suitable conditions for plant growth following LC and improved irrigation management. However, declines in vegetation health and moisture retention in May and June highlight ongoing water stress. Despite the positive effects of LC on improving irrigation infrastructure, further improvements in water management and sustainable practices are needed to fully alleviate moisture stress and ensure long-term agricultural sustainability. This study provides insight into the influence of LC on agricultural parcels and supports continued implementation of the GAP project to improve agricultural practices and water management in the southern Anatolia Region of Türkiye.

## Introduction

Population growth and global climate change have led to increased demand for agricultural production, requiring structural land arrangements and improvements in agricultural areas to meet the needs of the growing population (Giller et al., 2021; Jararweh et al., 2023). Efficient use of agricultural lands is also important for food security, environmental sustainability, and soil health (AbdelRahman, 2025). Land consolidation (LC) is an agricultural land arrangement that is implemented worldwide, especially in rural areas, and aims to increase the productivity of agricultural crops, optimize water use, and improve ecosystem services (Arslan et al., 2024; Uyan et al., 2015). Farmers often own parcels scattered across different village locations. This situation forces farmers to spend extra time traveling between parcels. Land fragmentation (LF), the division of large fields into smaller pieces, may occur due to inheritance or geographical conditions (Niroula & Thapa, 2005). Most of the agricultural land parcels fragmented into smaller areas are not used efficiently due to irrigation problems. Moreover, the division of agricultural lands into many small parcels can gradually cause the soil structure to deteriorate (Sklenicka, 2016). Furthermore, the number of planted areas decreases because corners of irregular parcels are unsuitable for planting because they hinder machinery use (Alturk, 2024). Therefore, LC regulations rearrange the physical structure of agricultural lands, allowing for the creation of larger and more regular parcels, which allows farmers to do more effective agriculture by providing more efficient use of water resources, preventing soil erosion, and reducing production costs (Bahar & Kirmikil, 2021). However, despite their advantages, LC regulations may sometimes negatively affect the socio-economic routines of small-scale farmers, for example, by causing them to share their consolidated larger land with the other shareholders (Van Dijk, 2007). Moreover, since LC processes convert many small agricultural land parcels into larger ones, this can cause biodiversity loss, ecosystem imbalance, and some changes in local vegetation (Yang et al., 2025).

The Southeastern Anatolia Project (GAP) is one of Türkiye’s largest regional development projects and was started by the government of the period in the 1980s. The project primarily aims to use water resources effectively, increasing agricultural production capacity and supporting economic development (Bilgen, 2020). People’s primary activities are agricultural activities and animal husbandry in the Southeastern Anatolia Region of Türkiye (Ceylan & Ozcelik, 2018; Ulaş, 2021). Many dams and irrigation channels have been built on the Euphrates (Fırat) and Tigris (Dicle) Rivers in the region within the scope of the GAP project (Khalil, 2023). The GAP project plays a supporting role in increasing the effectiveness of LC activities by improving the irrigation infrastructure in the region (Cetin, 2019). In parallel with the GAP project, LC works have been initiated to increase agricultural production and productivity in the region. LC applications are carried out by determining the soil quality with the grading method since the fragmented lands have different soil quality before LC (Tezcan et al., 2020). Türkiye is spending a large amount of money, time, and effort on LC projects every year to establish the infrastructure needed for agricultural lands (Aslan, 2021). On the other hand, GAP is not just an agricultural, irrigation, and energy project; it is a structural transformation project that may bring about cultural and social transformation in the Southeastern Anatolia Region of Türkiye (Gabrielyan, 2024).

Remote sensing satellite data are useful for examining vegetation and moisture changes resulting from irrigation infrastructure and LC activities worldwide (Hong et al., 2019; Naeem et al., 2022). In this paper, normalized difference vegetation index (NDVI), normalized difference moisture index (NDMI), land surface temperature (LST), soil moisture index (SMI), and modified normalized difference water index (MNDWI) were calculated from optical remote sensing satellite data as well as synthetic aperture radar (SAR) images due to their sensitivity to water. Several studies have used these spectral indices. Shan et al. (2019) compared the Remote Sensing Ecological Index (RSEI) and Ecological Index (EI) 6 years before and after LC and extended the spatial–temporal pattern study by applying the RSEI method to evaluate ecological environmental quality (EEQ) in a typical LC area in China. The results showed a strong comparability of RSEI and EI in ecological terms, with EEQ decreasing before and during LC and then increasing after LC. In another study, the effects of LC in the Chaohu Lake Basin on crop growth, soil moisture, and surface temperature changes were studied by Guo et al. (2020), using principal component analysis with four indices, namely NDVI, wetness index (WET), LST, and NDBSI. The study showed that LC improved the quality of the ecological environment. Moreover, the environmental impacts of conversion from agricultural lands to aquaculture along the Rasulpur River were examined by Mallick and Rudra (2021) using the NDVI, NDWI, MNDWI, soil adjusted vegetation index (SAVI), and LST spectral remote sensing indices, which were calculated from Landsat data between 2003 and 2017, and it was found that land use change caused environmental changes and they could be detected by spectral remote sensing indices. In addition to the previous study, Kocur-Bera and Małek (2024) calculated NDVI and SAVI to identify agricultural lands for consolidation using Sentinel-2 satellite images, while multispectral images of selected agricultural lands were obtained by an unmanned aerial vehicle (UAV) equipped with a multispectral camera. In this study, it was observed that multispectral images obtained using UAVs gave better results than images obtained from Sentinel-2 satellites, while the accuracy of NDVI and SAVI values was found to be low in small agricultural land parcels. Moreover, Yilgan et al. (2025) evaluated the effects of LC in agricultural soil blocks of the Czech Republic after one of the EU regulations applied to large agricultural land parcels that prohibits single crops up to 30 hectares. NDVI, NDMI, LST, and SMI were analyzed to observe changes on large agricultural land parcels using Landsat-8 remote sensing data between 2019 and 2021. Results showed that the strong positive correlation between NDVI and NDMI increased from *r* = 0.80 in 2019 to 0.92 in 2021, suggesting that productivity and soil degradation depend on plot size.

In addition to worldwide research, several studies were conducted using spectral remote sensing indices in Mardin in the southeastern Anatolia Region. Kiliçaslan et al. (2024) calculated SMI from Landsat-8 data and VV polarization of Sentinel-1 SAR images in Artuklu and Kızıltepe Districts of Mardin between June and September 2021 by using the Google Earth Engine (GEE) cloud-based platform. Results were compared with soil temperature and moisture values measured with ground devices in 27 cotton parcels in 8 villages in Artuklu and Kızıltepe. In the study, the coefficient of determination was *R*^2^ = 0.67 for Sentinel-1 VV polarization and *R*^2^ = 0.85 for Landsat-8 SMI. Moreover, Dursun et al. (2025) analyzed changes in plant and soil moisture and LST changes on agricultural land parcels between 2013 and 2021 in two villages of Mardin, Türkiye, using Landsat-8 remote sensing data. The study compared NDVI, NDMI, LST, and SMI over time and found significant decreases in 2021.

The literature review indicates that no studies in the Southeastern Anatolia Region of Türkiye comprehensively address the effects of the GAP project and LC activities on land structure and agricultural activities combining optical and radar satellite images, especially those covering the recent past years, e.g., 2023 and 2024. This study fills a gap in the literature by comparing the Sentinel-1 with Landsat 8/9 spectral indices in southeastern Anatolia, covering the last 10 years, including 2023 and 2024. GEE brings a different methodological perspective to the study. The aim of this study is to observe changes in four villages of southeastern Anatolia pre-LC and post-LC with 10 years of coverage of satellite data between 2015 and 2024. The study is also important to observe the effects of the recent LC carried out in the southeastern Anatolia Region in 2019. The paper highlights GAP project effects in southeastern Anatolia. Moreover, it also sheds light on agricultural improvements and is expected to be helpful for land management projects as well as soil scientists to create future scenarios related to soil health and degradation. Furthermore, the results are expected to encourage new regulations for soil health, water efficiency, and protection of agricultural lands, allowing for the creation of sustainable agricultural policies and soil protection laws. In this context, Sentinel-1 SAR data were filtered to obtain VV polarization backscattering values. NDVI, NDMI, and MNDWI spectral remote sensing indices were calculated from Landsat 8/9 data over 10 years, separated into pre-LC (2015–2019) and post-LC (2020 –2024). The mean values of pre-LC and post-LC were calculated, and thus the difference between pre- and post-mean values has provided changes of spectral remote sensing indices in the region. The same steps were applied to Sentinel-1 SAR backscattering. Finally, the relationships between the changes in the indices and the SAR changes were investigated by computing Pearson’s correlation coefficients.

## Materials and methods

### Study area and datasets

**Fig. 1:**
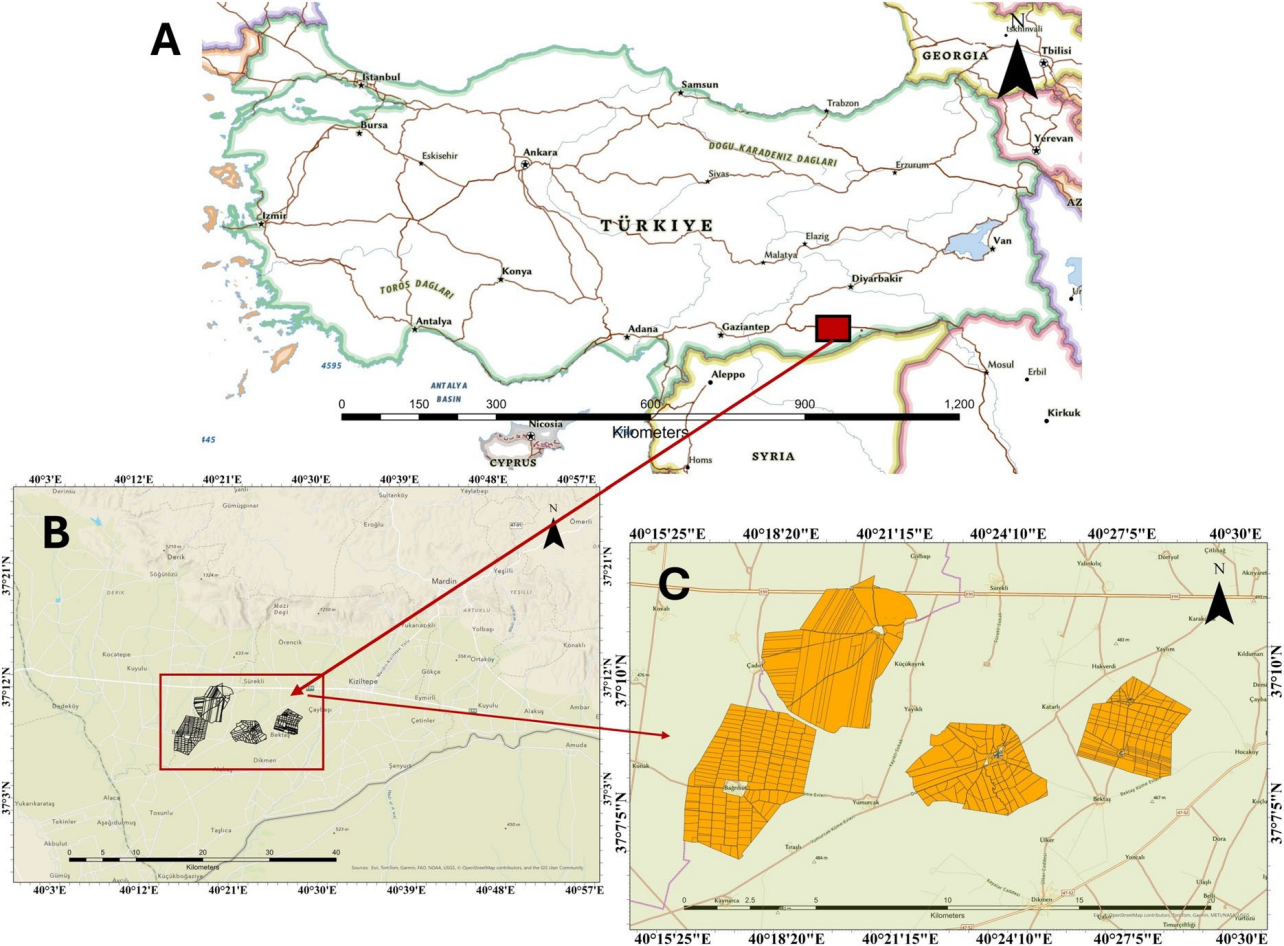
Study area: **A** location of the study area in Türkiye, **B** study area in the Southeastern Anatolia Region, and **C** agricultural land parcels in the study area

The study area is shown in Fig. 1C, covers four different villages, which are Bağrıbütün, Başak, Büyükayrık, and Demirci, selected in the Kızıltepe Plain of the Southeast Anatolia Region of Türkiye with coordinates between 37° and 38° N latitude and 40° and 41° E longitude. Kızıltepe is a town in the Mardin metropolitan city, and the total area of agricultural land parcels in the four villages is 61.2 km^2^. The study area, where important civilizations have lived, has semi-arid characteristics and has had dry farming since ancient times. The region was waiting for irrigation management under the GAP project for a long time (Yenigun et al., 2023). Moreover, the Kızıltepe Plain enables agricultural activities in the region with its large irrigation areas fed by the branches of the Euphrates River, and thanks to the arable alluvial soils, products such as wheat, cotton, lentils, and chickpeas are grown (Kılıçaslan, 2024). However, summer seasons are hot and dry, while winters are cold and rainy, and air temperatures can often reach 40 °C during the summer, while they are low in winter in Mardin (Hadrovic, 2024). Since the average annual rainfall is limited in the region, agricultural irrigation is important for the sustainability of agricultural activities in the region (Kartal et al., 2024).

Data selections were done based on the season of agricultural activity in the study area. The months of April, May, and June where the period is characterized by intense agricultural activities in the region. Thus, Sentinel-1 SAR and Landsat 8/9 operational land imager (OLI) satellite images (Landsat Collection 2 Level 2 SR) were obtained for April, May, and June with a 10-year range between 2015 and 2024. The Landsat-8/9 data were selected with less than 30% cloud coverage, and the Landsat-8/9 series has 30 m spatial resolution, excluding two thermal bands of the satellite, while the Sentinel-1 SAR data has 10 m spatial resolution. Sentinel-1 SAR data were selected for interferometric wide (IW) beam mode and VV polarization due to high detectability of sparse and low-density agricultural crops. The shapefile of agricultural land parcels was obtained from the Şanlıurfa State Hydraulic Works, 15th Region Directorate. The study region has been known for its agricultural activities for many years. Only the types of crops planted vary. However, we do not have detailed information on which crops were planted in which plots in which year. According to climate data that was obtained from (https://weatherandclimate.com/turkey/mardin/kiziltepe), the 5-year average air temperature of April between 2015 and 2019 is 17.4 °C while it is 24.1 °C in May and 31.2 °C in June. In addition to this, average temperatures in recent years for April, May, and June are 17.8 °C, 24.3 °C, 31.5 °C, respectively. Furthermore, monthly mean temperature over years obtained by using “temperature_2m” band from ERA5 dataset that is produced by the Copernicus Climate Change Service (C3S) (Hersbach et al., 2020). The ERA5 has 0.1° × 0.1° spatial resolution, which is approximately 9 km × 9 km at the equator (Muñoz-Sabater et al., 2021). The monthly mean precipitation data was obtained from Climate Hazards Group InfraRed Precipitation with Station data (CHIRPS) which has an 0.05° × 0.05° degree spatial resolution (Funk et al., 2015). Moderate Resolution Imaging Spectroradiometer (MODIS) LST data that has 1 km spatial resolution also were calculated to check the partial correlation coefficient between surface temperature and precipitation.

### Methodology

**Fig. 2:**
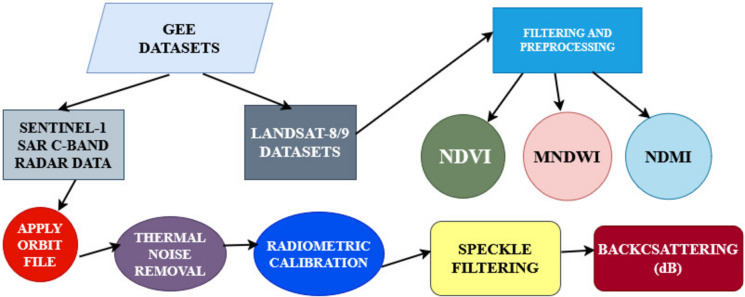
Flowchart of methodology steps

Monthly changes of Sentinel-1 SAR backscattering and different spectral remote sensing indices, which are NDVI, NDMI, and MNDWI, were calculated for the southeastern Anatolia Region of Türkiye using the GEE cloud-based platform for April, May, and June. The GEE is an essential tool for computation of huge datasets, and it provides different satellite data for various environmental analyses (Gorelick et al., 2017). Methodology steps of image processing and index calculations are shown in Fig. 2. Monthly mean pixel values from satellite images were calculated from 2015 to 2019 and from 2019 to 2024. On the other hand, the average pixel values of the satellite images were taken for pre-LC and post-LC because of LC activities that were totally done in those four villages in 2019. The difference between these pixel values represents the change.

### Pre-processing of Sentinel-1 synthetic aperture radar (SAR)

The Sentinel-1 SAR images in “COPERNICUS/S1_GRD” collection have several noise and radiometric problems. One important problem caused by the satellite's electrical system is thermal noise. Thermal noise affects SAR images by producing a low backscattering intensity effect of energy (Mullissa et al., 2021). The satellite’s position and velocity are saved in an orbit file, which must be used to improve the accuracy of SAR photos. Thermal noise removal was applied by the Sentinel-1 toolbox in GEE and border noise removal was applied to the satellite images to eliminate border artifacts using the formula that is given in Eq. (1).

1 $${\mathrm{G}}_{inner} =\mathrm{I}-{\Delta br})$$

where I represent original image footprints, Δbr is border reduction while inner buffer geometry is *G*_inner_.

Radiometric calibration is required to convert the raw digital numbers (DNs) of SAR images into meaningful physical pixel units such as the radar backscatter coefficient, radiometric. This calibration provides SAR data to be compared with other quantitative measurements, and the formula is given in Eq. (2) (Wyatt, 2012).

2 $$\upsigma 0=\text{K }\times {DN}^{2}$$

where DN is a digital number of SAR images while σ0 is original backscattering values. K is the absolute calibration factor. In addition, original radar backscatter coefficients were converted to decibels (dB) using the formula given in Eq. (3).

3 $${\upsigma 0}_{\mathrm{dB}}=10 \times {\mathrm{log}}_{10} (\upsigma 0)$$

where σ0 is the original backscattering coefficient while σ0_dB_ is the backscattering coefficient in decibels.

Terrain correction improves accuracy and eliminates distortions by providing the backscattering pixels of SAR photos corresponding with their actual coordinates on the Earth’s surface in SAR photographs (Loew & Mauser, 2007). The GEE cloud-based platform uses a digital elevation model (DEM) that was obtained from Advanced Spaceborne Thermal Emission and Reflection Radiometer (ASTER) for application of terrain correction. The correction was done using incidence angle and slope by the formula which is given in Eq. (4).

4 $$0{^{\prime}} =\upsigma 0 \times \mathrm{cos}({\uptheta }_{incidence} )\times \frac{cos({\theta }_{incidence})}{cos({\theta }_{incidence \,corrected})}$$

where 0′ is the corrected backscattering coefficient σ0 is the original backscattering coefficient. θ_incidence corrected_ is a corrected incidence angle while θ_incidence_ is the original incidence angle.

Lee speckle filtering was applied to satellite images to remove noise in synthetic aperture radar (SAR) images, improving interpretation and radiometric quality. The formula of Lee filtering is given in Eq. (5) (Lee, 1980).

5 $${\mathrm{I}}_{F} (\mathrm{x},\mathrm{y})={\mathrm{I}}_{M}(\mathrm{x},\mathrm{y})+\mathrm{k}(\mathrm{x},\mathrm{y})\cdot (\mathrm{I}(\mathrm{x},\mathrm{y})-{\mathrm{I}}_{M} (\mathrm{x},\mathrm{y}))$$

where k is the damping factor while I_*M*_ is the local mean. In addition, *I* represent the original pixel value and I_*F*_ is the filtered pixel value.

### Normalized difference vegetation index (NDVI)

The NDVI is a remote sensing index that discriminates soil and vegetation from each other, and it was calculated by using near-infrared and red bands of surface reflectance data, and the calculation formula is given in Eq. (6) (Tucker, 1979):

6 $$\text{NDVI }=\left(\text{NIR }-\text{ R}\right) / (\text{NIR }+\text{ R})$$

where NIR and R represent the near-infrared and red bands of the satellite data, respectively. Values that are closer to 1 represent high vegetation density, while values that are closer to −1 represent non-vegetation.

### Modified normalized difference water index (MNDWI)

MNDWI is a modified version of the normalized difference water index (NDWI) and is mostly used to detect open water and flooded areas, as well as in drought monitoring of semi-arid and arid regions. Also, it is used for the enhancement of open water features, and it is more suitable for extracting water information for a region because of its advantage in reducing and removing land noise over the NDWI, calculated by using a formula that is given in Eq. (7) (Xu, 2006).

7 $$\text{MNDWI }= \frac{(G - SWIR)}{(G + SWIR)}$$

where G is the green band, while SWIR is the shortwave infrared band of the satellite.

### Normalized difference moisture index (NDMI)

NDMI has an interval between −1 and 1 that provides information about canopy moisture levels, and values near to 1 represent higher moisture levels while the values near to −1 represent drought conditions (Niccoli et al., 2024). The NDMI is calculated using near-infrared and short-wave infrared bands of surface reflectance data of Landsat satellite images. The formula that is given in Eq. (8) by Wilson and Sader, (2002) is used to calculate NDMI values.

8 $$\text{NDMI }=(\mathrm{NIR}-{\mathrm{SWIR}}_{1})/(\text{NIR }+ {\mathrm{SWIR}}_{1})$$

where NIR is the near-infrared band, SWIR_1_ is the first short-wave infrared band of the Landsat-8/9 satellite, and the wavelength of the band is between 1.57–1.65 μm.

### Statistical evaluation of datasets

The Pearson’s correlation coefficients (r) between some spectral remote sensing indices with SAR data were checked for better understanding of changes on pre and post LC using 944 points which are distributed homogeneously in the study area. Selection of pixel points was chosen as stratified that are equally distributed to every parcel in the study area. Fishnet grids provide equal distance between selected points, and it creates the spatially homogeneous pixel samples, ensuring distribution and minimizing spatial sampling bias. Since the 944-pixel data in this study were collected at specific spatial intervals, and assuming that different agricultural crops are cultivated in the region, the likelihood of spatial autocorrelation is very low. The Pearson correlation coefficient measures the strength and direction of the linear relationship between two variables (Schober et al., 2018). While the correlation coefficient varies between − 1 and + 1, − and + indicate the direction of the relationship, and as it approaches − 1 and 1, it is seen that there is a strong relationship between the two data (Ratner, 2009). P-values were calculated to assess whether the observed correlations were statistically significant for better understanding the statistical significance of the relationships between the indices. Correlations with *p* < 0.05 were considered statistically significant. The statistical evaluations were done by using Statistica v13 (TIBCO Software Inc., USA) software.

## Results

**Fig. 3:**
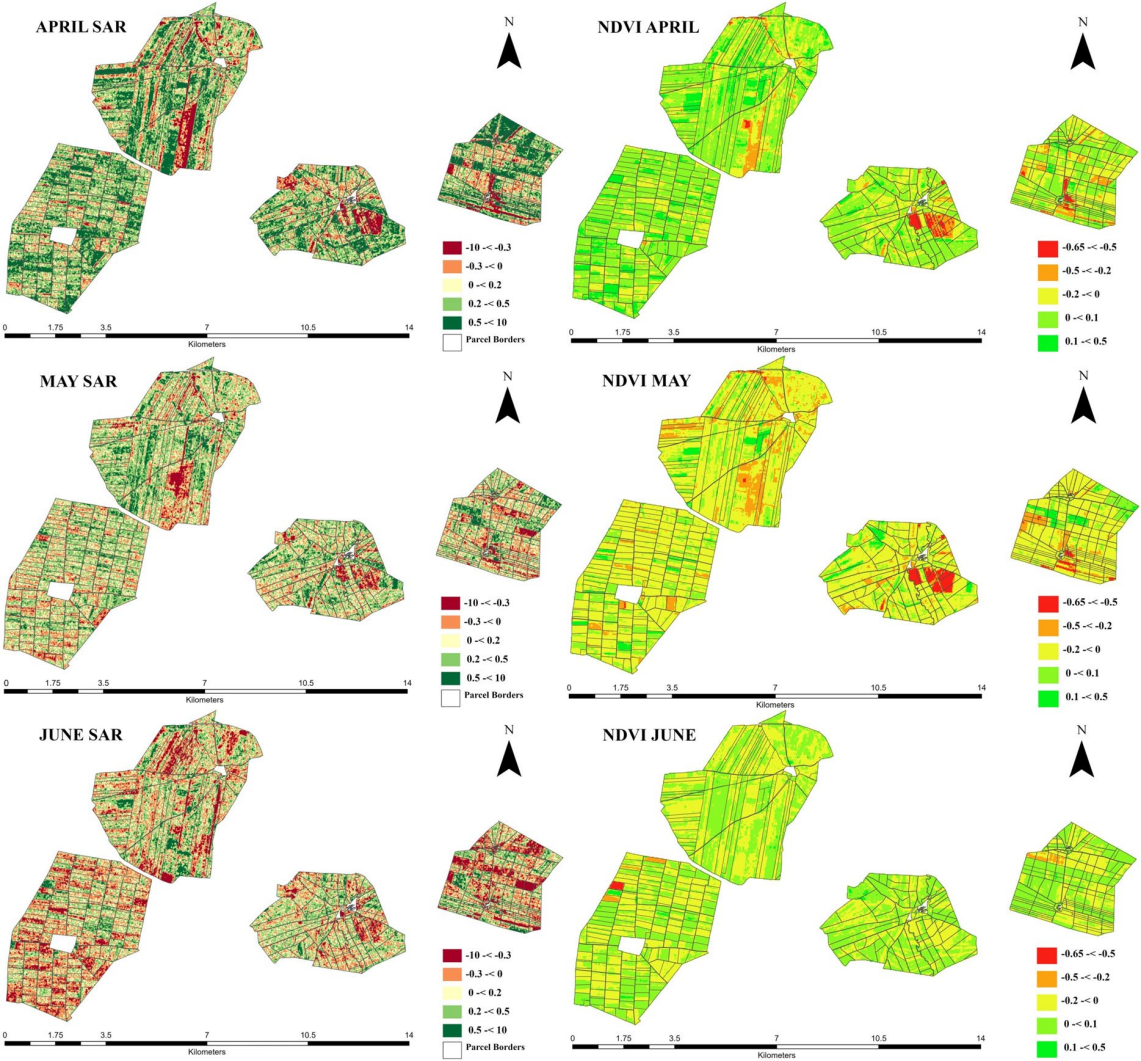
Change detection maps of ΔSAR and ΔNDVI distribution during agricultural season

Changes in vegetation density and crop moisture on land parcels were observed in four villages in the Southeast Anatolia Region of Türkiye using remote sensing Sentinel-1 SAR and Landsat 8/9 datasets before and after 2019. The change detection maps of ΔSAR and ΔNDVI are shown in Fig. 3 for April, May, and June. Sentinel-1 SAR data showed an increase in April while decreasing gradually in May and June compared to pre-LC. ΔNDVI also showed a significant April increase, similar to the Sentinel-1 SAR trend. However, a decrease of ~ 0.2 was observed in most plots in May. While Sentinel-1 SAR shows a decrease in June compared to pre-LC, the ΔNDVI shows an increased distribution in June compared to May and a decrease compared to April.

**Fig. 4:**
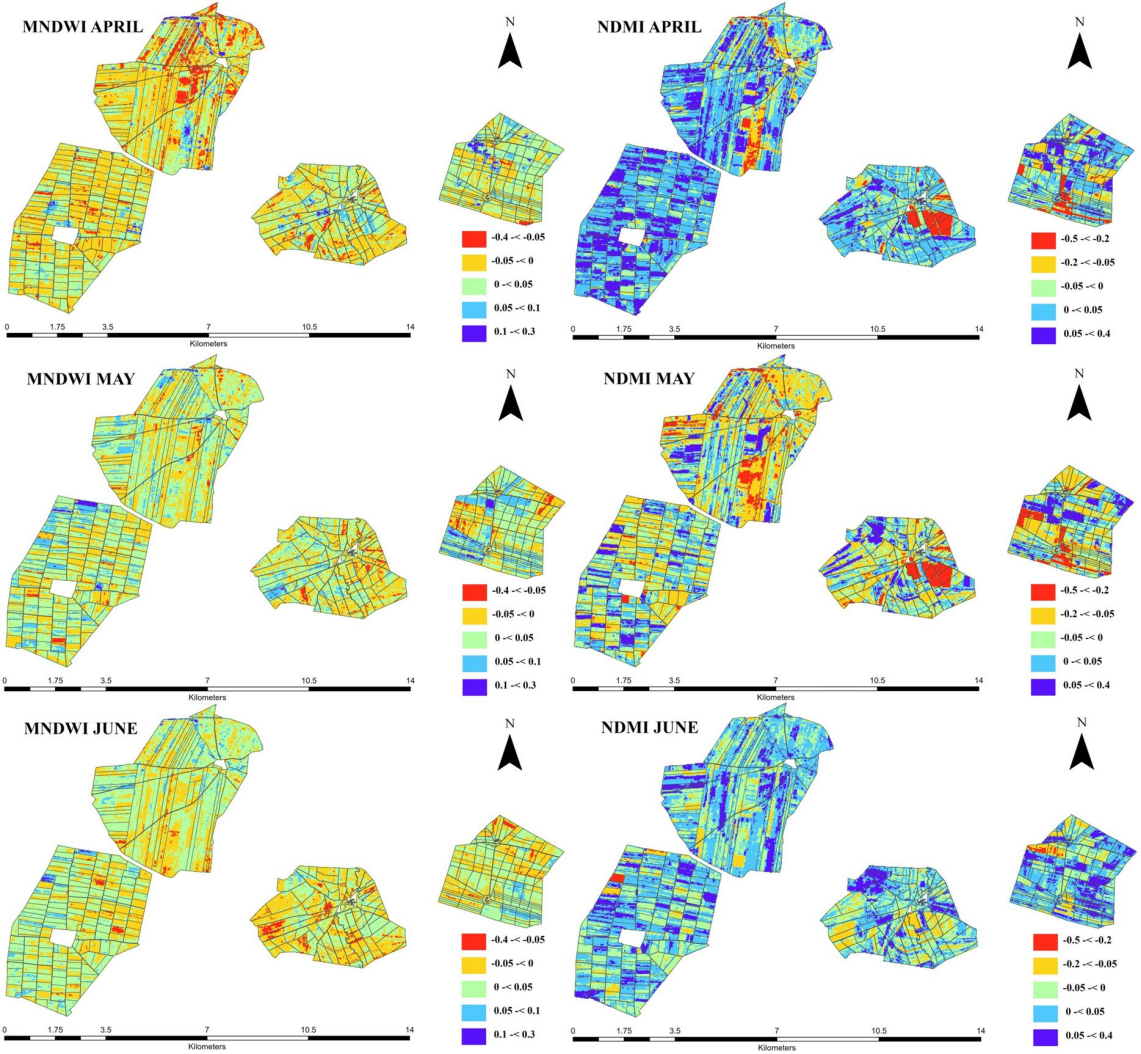
Change detection maps of ΔMNDWI and ΔNDMI distribution during agricultural season

The ΔMNDWI and ΔNDMI changes in agricultural land parcels are shown in Fig. 4 for 3 months (April, May and June). ΔMNDWI and ΔNDMI exhibit distinct temporal changes reflecting the effects of LC activities and seasonal changes in surface water and crop moisture conditions. According to the results, ΔNDMI has been increasing in April while ΔMNDWI was decreasing in the study area. The same decreasing trend of ΔMNDWI was also observed in May and June, but ΔMNDWI decreased the most in April. The ΔNDMI increased the most in April, and a significant decline in ΔNDMI was seen in May. The ΔMNDWI and ΔNDMI indices demonstrated a clear temporal contrast between the early and late months of the growing period.

The mean values of changes in spectral indices (ΔSAR, ΔNDVI, ΔMNDWI, ΔNDMI) are given in Table 1 with their standard deviation for April, May, and June respectively. Mean values showed declines in June, with higher values in April compared to other months. Also, ΔNDVI increased slightly in May (0.019 ± 0.05) and remained nearly constant in June (− 0.001 ± 0.02). In addition, ΔMNDWI and ΔNDMI showed minor changes; a slight decrease was observed in June for ΔMNDWI, reaching (−0.017 ± 0.02), and for ΔNDMI, reaching (− 0.006 ± 0.02). Moreover, ΔSAR values showed the highest increase in April and stabilized in May and June, decreasing to (0.24 ± 0.71) and (0.19 ± 0.77), respectively.

**Table 1:** Mean values of changes of spectral indices and their standard deviations (SD) over 3 months

Mean values	ΔSAR	SD	ΔNDVI	SD	ΔMNDWI	SD	ΔNDMI	SD
April	1.03	*0.96*	0.007	*0.05*	0.005	*0.03*	0.015	*0.04*
May	0.24	*0.71*	0.019	*0.05*	− 0.009	*0.03*	0.019	*0.05*
June	0.19	*0.77*	− 0.001	*0.02*	− 0.017	*0.02*	− 0.006	*0.02*

Confidence intervals were calculated to understand the probability distribution of changes in vegetation, soil moisture, water surface, and radar backscatter. According to the percentage values shown in Table 2, changes in ΔNDVI, ΔNDMI, and ΔMNDWI are generally not very significant, with values between P70 and P95 being (from 0.013 to 0.084), (from 0.019 to 0.077), and (from 0.018 to 0.043), respectively. On the other hand, ΔSAR values ranged between 0.207 and 0.34, and more pronounced changes were observed in radar data than in optical data due to the surface structure and moisture sensitivity.

**Table 2:** Distribution of parcel level changes (ΔNDVI, ΔNDMI, ΔMNDWI, and ΔSAR) across selected percentiles

Percentile	ΔNDVI	ΔNDMI	ΔMNDWI	ΔSAR
P70	0.013	0.019	0.018	0.207
P80	0.030	0.038	0.026	0.245
P90	0.055	0.064	0.035	0.294
P95	0.084	0.077	0.043	0.341

**Fig. 5:**
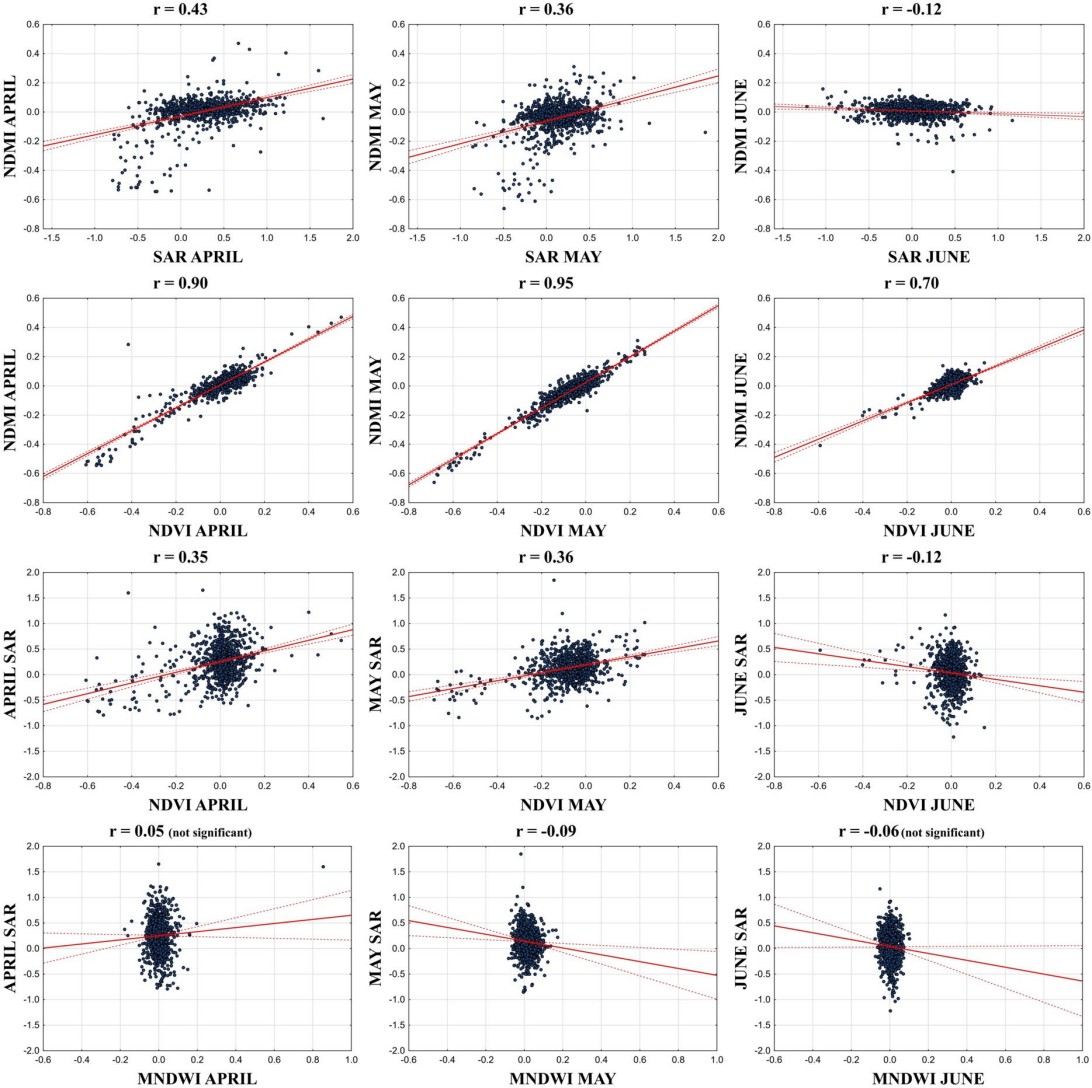
Scatterplots of statistical analysis between Sentinel-1 SAR data and spectral indices

The Pearson’s correlation relationships of spectral indices and Sentinel-1 SAR backscattering data are shown in Fig. 5 as scatterplots. Correlations between index changes and Sentinel-1 ΔSAR data illustrate LC effects on plots.

According to the scatterplots, the highest correlation coefficient between changes of indices was found between ΔNDVI and ΔNDMI in May, while the lowest correlations were between ΔMNDWI and Sentinel-1 ΔSAR. In addition, a strong positive correlation was observed between changes of ΔNDVI and ΔNDMI in all months, but the lowest correlation coefficient was *r* = 0.70 in June. Moreover, the correlation of the change of ΔNDMI with the change of ΔSAR data was positive in April and May, while a weak negative correlation coefficient of *r* = −0.12 was observed in June. A similar trend was also observed between the change of ΔNDVI and the change of Sentinel-1 SAR data. Furthermore, the only significant weak negative correlation was between ΔMNDWI and ΔSAR (*r* = −0.09) in May. SAR–MNDWI correlations in April and June were not significant (*p* > 0.05) and thus unreliable.

**Fig. 6:**
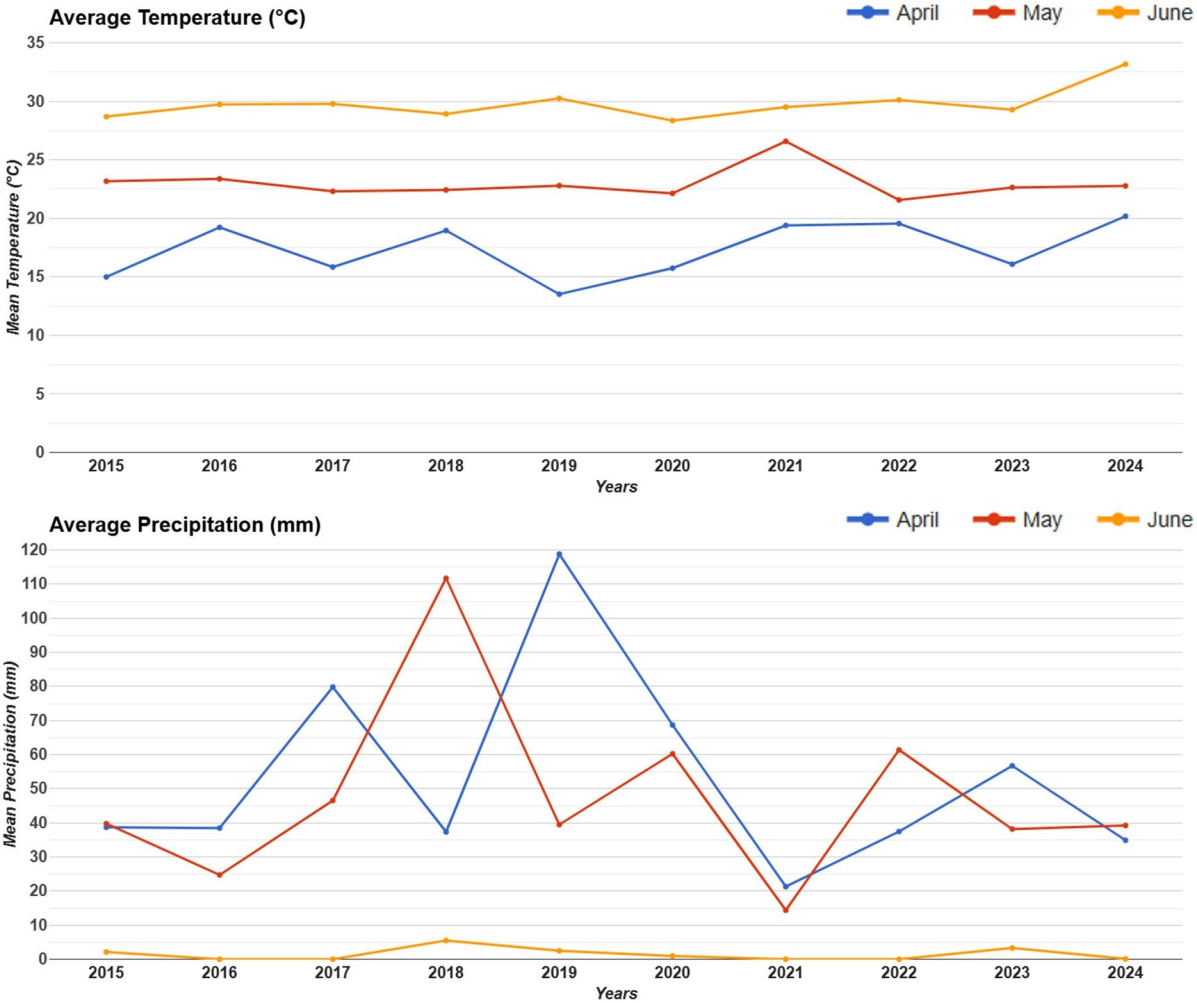
Temperature and precipitation graphs of study area by months and years

The average air temperature and precipitation for 3 months (April, May and June) are shown in Fig. 6. The lowest average precipitation and the highest average temperature were observed in June for every year. The lowest precipitation was in June around 5 mm every year compared to other months. In addition, mean air temperature ranged from 14 °C in April 2019 to 32 °C in June 2024. Moreover, the average temperature was higher in May compared to April while there are fluctuations on the average precipitation in April and May for some years. The highest mean precipitation was in April 2019 as 120 mm while the lowest was in May 2021 as 15 mm as well as it was also lower in April 2021 as 20 mm. The average precipitation was also higher in May 2018 approximately 110 mm. Moreover, partial correlation between MODIS LST and CHIRPS precipitation was −0.75.

## Discussion

Changes in vegetation cover and moisture retention of agricultural crops in agricultural land parcels belonging to four villages in the Kızıltepe District of Mardin Province, located within the GAP project area where LC work has been carried out, were analysed using Landsat-8/9 and Sentinel-1 SAR satellite images. Changes between 5-year averages of NDVI, NDMI, and MNDWI before and after 2019 were calculated from Landsat 8/9 satellite images, while the Sentinel-1 SAR change was also calculated to obtain more precise results. Land consolidation is widely implemented as a policy tool in many countries around the world (Liu et al., 2020; Sklenicka, 2006). Most previous studies in the world have examined some remote sensing indices separately, such as NDVI and NDMI to observe surface moisture and vegetation cover to find possible effects of land use planning and land consolidation (Jindo et al., 2021; Yang et al., 2025; Yilgan et al., 2025). Additionally, some studies performed analyses using NDVI and NDWI indices by comparing them to microwave and SAR data (Holtgrave et al., 2020). In addition to studies around the world, water availability, land use, and soil structure were examined using remote sensing indices in previous studies conducted in the GAP region (Dursun et al., 2025; Karabulut et al., 2023; Özcan et al., 2018). Thus, conducting the analysis by integrating optical and microwave datasets gives the study an important meaning for the analysis in the GAP region, and the study fills an important gap in the literature because there is no comprehensive analysis conducted with these data in the GAP region, particularly by integrating MNDWI. This study is the first integrated multi-sensor analysis using NDVI, NDMI, MNDWI, and SAR in the GAP region.

Significant positive changes in Sentinel-1 SAR and ΔNDVI in April indicate more suitable conditions for plant development occurred at the beginning of the agricultural season after 2019 compared to before 2019. A similar increase is also observed in ΔNDMI in parallel with this development of vegetation cover after 2019. Thus, soil organic matter, which influences plant growth and water retention, may have been positively affected by LC in agricultural parcels, while the region may also have experienced sufficient seasonal rainfall during the spring period. Similarly, a study conducted by Pijanowski et al. (2022) in Poland indicated that LC affects the water retention of the soil, thus alleviating drought and flood problems. Furthermore, Chen et al. (2022) demonstrated that when vegetation productivity and soil productivity were compared, soil productivity showed a stronger and more positive response to LC and SOM increased agricultural land productivity under the influence of LC. In contrast, decreases in ΔNDVI, ΔNDMI, and Sentinel-1 ΔSAR in May and June may indicate that vegetation is weakening due to moisture stress and decreasing amounts of rainfall. Especially in May, the decrease in ΔNDVI and ΔNDMI by about 0.2 in most agricultural plots may be due to factors such as soil moisture depletion or water deficit. Taking into account the CHIRPS precipitation graph shown in Fig. 6, the fact that the amount of precipitation in May and June is quite low also supports this situation. Nega et al. (2023) also observed that there was a spatial and direct relationship between soil moisture and vegetation cover in a semi-arid area, indicating that areas with high soil moisture had higher vegetation cover. However, ΔNDMI’s peak in April followed by a sharp May decline indicates that the moisture in the soil and plants decreases and that the plants are under water stress compared to April. In addition, Imtiaz et al. (2024) calculated NDVI and NDMI indices using Landsat-8 and MODIS satellite images to analyse LST and SMI for agricultural soil plots using the cloud-based GEE platform. The study showed a strong positive correlation (≥ 0.95) between NDVI and NDMI, similar to the findings of this study. The strong positive relationships of NDVI with NDMI were also shown by Ghasemloo et al. (2022), in an agricultural farm area using Landsat-8 remote sensing data.

ΔMNDWI increased in April but decreased in May and June, indicating that water retention in plants and soil is higher at the beginning of the season and decreases over time. However, it should be noted that MNDWI is generally used to detect water in open areas or flood areas (Öztürk & Sesli, 2015), and its use for agricultural activities is limited. In the study examining water and moisture indices, Khalid et al. (2021) indicated that NDMI gave better results than MNDWI. Moreover, in June, ΔSAR decreased compared to May, while ΔNDVI increased, suggesting that ΔNDVI reflects changes in plant health and that ΔNDVI change is more sensitive to plant health than changes in ΔSAR data. SAR data can better detect moisture changes due to its sensitivity to water, while ΔNDVI can provide a better interpretation of vegetation density and plant health. Torres-Quezada et al. (2025) showed that vegetative growth stages can be effectively observed with NDVI and SAVI. Furthermore, the increase observed in NDVI and NDMI changes in June and the decrease in SAR changes in June highlight the water availability in agricultural plots. In support of this situation, Ibrahim et al. (2023) demonstrated that NDVI and NDMI are sensitive to moisture using remote sensing data. In another study, drought analysis was conducted by Gumus et al. (2021) using monthly total precipitation data obtained from 15 stations in nine different cities in the GAP project area, and according to the spatial analysis results, a decreasing trend in drought was also observed in most of the region at almost all the time scales.

Strong positive correlations between ΔNDVI and ΔNDMI in May demonstrate sensitivity to similar environmental factors, while the weak negative correlation between ΔMNDWI and Sentinel-1 ΔSAR reveals that SAR data cannot detect moisture changes very well in all periods. Ma et al. (2022) indicate that SAR data generally exhibit a positive correlation with NDWI, but VH polarization has a higher correlation with NDWI than VV polarization. However, in our study using VV polarization, the correlation between ΔMNDWI and ΔSAR was observed only positive in April and negative in May and June. This suggests that MNDWI-SAR relationships vary with climate, seasonality, and SAR polarization type. In addition, the decline in ΔNDVI–ΔNDMI correlation to *r* = 0.70 in June indicates that there is a decrease in vegetation cover and accordingly in the moisture content of the plants, while the decrease in the correlation relationship of ΔNDMI and ΔNDVI with Sentinel-1 ΔSAR changes as *r* = − 0.12 seen in June also proves that the available moisture content cannot be used efficiently by plants in agricultural plots in June. Previous studies have shown strong positive correlations between SAR consistency and NDVI in agricultural areas (Villarroya-Carpio et al., 2022), supporting the use of SAR in vegetation monitoring. However, despite positive correlations in April and May, the negative correlation (− 0.12) in June reflects seasonal moisture stress and reduced water availability, consistent with the known sensitivity of SAR and optical indices to soil and plant water content.

Summary, remote sensing enables rapid monitoring and provides precise imagery at both small and large scales (Sandau et al., 2010). In addition, the combined use of Sentinel-1 SAR and spectral remote sensing indices is a powerful method to better understand vegetation changes, moisture dynamics, and crop estimation in agricultural lands (Narin et al., 2025). Furthermore, combining Analytic Hierarchy Process (AHP), Geographic Information System (GIS), and remote sensing indices allows for a better analysis of land suitability (AbdelRahman et al., 2025).

Thus, analysis with integrated remote sensing data increases the reliability of the results. In addition, SAR data is more responsive to surface conditions than other indices, providing more precise results. Single-index NDVI analyses show limited effectiveness of land consolidation in increasing productivity (Jin et al., 2017). Positive changes in April suggest that LC improved plant growth and moisture retention. However, the decline in vegetation health and moisture retention during the later month in June indicates the vulnerability of the region to moisture stress, exacerbated by reduced rainfall. On the other hand, the May decline indicates that GAP project effects are not yet evident in this area, while some degradation in the soil structure may have prevented crops from using water efficiently in June. Moreover, the confidence intervals for change estimates presented in Table 2 indicate that change is more intense in some plots and that spatial heterogeneity exists. They also reveal that environmental changes in the study area are measurable but generally small in scale. In parallel with this situation, Hong et al. (2019) showed that the effect of LC on agricultural production varies by region; these changes can be associated with the properties of soil and water, as well as differences in climatic conditions. In support of the study results, drought analysis was conducted in all GAP regions by Gumus et al. (2021), the observed significant decreasing drought trend is mostly located in regions close to the Syrian border where agricultural activity is high and cover our study area. Thus, the positive effects of the GAP Project and land consolidation activities in the GAP region are also revealed by the drought analysis study. Moreover, it was demonstrated by Molnárová et al. (2023) that the outcomes of land consolidation are highly dependent on the structure of the consolidation projects. Therefore, LC efforts undertaken in every country do not result in the same efficiency.

In addition, Chen et al. (2022) demonstrated that vegetation and soil responded differently to LC applications, and that LC application increased soil fertility more than vegetation fertility. Although increased LC applications support agricultural development, problems are also encountered during implementation. The problems faced by farmers during the implementation of LC projects were examined by Tunali (2024), and the study showed that farmers’ education, land holdings, and age significantly influenced their views on LC projects. Considering all the information, the study results indicate the positive effects of LC and that the GAP project should affect more areas, thus supporting policymakers in addressing deficiencies and ensuring sustainable agriculture. Overall, the study highlights spatial heterogeneity and seasonal dynamics in soil moisture and vegetation cover, providing valuable input for modeling LC impacts and guiding future GAP-related policy and management decisions. It also suggests improvements in irrigation and soil management.

### Limitations of the study

MNDWI was developed for open water detection, making it less sensitive to vegetated surfaces. The weak correlation between MNDWI and SAR observed in this study may be related to the limited suitability of MNDWI for agricultural moisture monitoring. In addition, seasonal and annual variations were not assessed because the analyses focused on April, May, and June, representing the main growing season, limiting the generalizability of the findings to the entire agricultural season. Moreover, CHIRPS precipitation data provide general information, but the lack of precise irrigation schedules or local meteorological measurements limits a deeper understanding of how water management practices affect observed vegetation and soil moisture changes. Furthermore, it is not known in detail which agricultural crops were planted in which years in the agricultural soil plots, and crop composition change analysis could not be performed, limiting the reliability of the results. Further investigation of land use, crop composition, and vegetation productivity is needed to estimate the impact of LC projects on micro level yield changes (Demirdogen, 2025). In addition, direct field observations are not available, and soil quality was not assessed in the study area. Thus, validation and calibration using rainfall or irrigation records were not possible.

## Conclusion

This study used an integrated multisensory approach, combining Landsat-8/9 NDVI, NDMI, and MNDWI with Sentinel-1 SAR data to monitor vegetation dynamics and water availability in agricultural lands in Bağrıbütün, Başak, Büyükayrık, and Demirci villages in Kızıltepe District in the GAP region. The study results demonstrated that LC may positively impact early season vegetation growth and moisture, contributing to increased agricultural productivity under suitable rainfall conditions. In addition, the study highlighted the limitations of LC under late season water and moisture stress. Moreover, the study showed that aggregation effects can be observed by integrating indices, and plant development in the GAP region also depends on climate parameters. This information enhances knowledge about the effectiveness of LC in semi-arid regions and provides a framework for evaluating land management practices using an integrated remote sensing method. In summary, the GAP and LC projects play a key role in agricultural sustainability. However, further improvements in water management and plant health are needed. Additionally, further improved irrigation infrastructure and more effective water resources management are needed for sustainable agricultural practices. Furthermore, data integration opens opportunities for future research, including more detailed crop composition analyses, long-term monitoring across all agricultural cycles, and incorporating local irrigation and soil quality data to better understand LC impacts. This approach can guide sustainable agriculture policy by identifying areas for targeted interventions to optimize water use and crop yields.

## Data Availability

All data generated or used during the study appear in the submitted article.
